# Optical Fibre Sensors Using Graphene-Based Materials: A Review

**DOI:** 10.3390/s17010155

**Published:** 2017-01-14

**Authors:** Miguel Hernaez, Carlos R. Zamarreño, Sonia Melendi-Espina, Liam R. Bird, Andrew G. Mayes, Francisco J. Arregui

**Affiliations:** 1 School of Chemistry, Faculty of Science, University of East Anglia, Norwich Research Park, Norwich NR4 7TJ, UK; Andrew.Mayes@uea.ac.uk; 2 Department of Electrical and Electronic Engineering, Universidad Publica de Navarra, Pamplona 31006, Spain; parregui@unavarra.es; 3 Institute of Smart Cities, Universidad Publica de Navarra, Pamplona 31006, Spain; 4 School of Mathematics, Faculty of Science, University of East Anglia, Norwich Research Park, Norwich NR4 7TJ, UK; s.melendi-espina@uea.ac.uk (S.M.-E.); Lauren.Bird@uea.ac.uk (L.R.B.)

**Keywords:** optical fibre sensors, graphene, graphene oxide, reduced graphene oxide, carbon materials, thin films, nanostructured coatings

## Abstract

Graphene and its derivatives have become the most explored materials since Novoselov and Geim (Nobel Prize winners for Physics in 2010) achieved its isolation in 2004. The exceptional properties of graphene have attracted the attention of the scientific community from different research fields, generating high impact not only in scientific journals, but also in general-interest newspapers. Optical fibre sensing is one of the many fields that can benefit from the use of these new materials, combining the amazing morphological, chemical, optical and electrical features of graphene with the advantages that optical fibre offers over other sensing strategies. In this document, a review of the current state of the art for optical fibre sensors based on graphene materials is presented.

## 1. Introduction

Graphene (G), a two-dimensional carbon material with one-atom-thickness, has become a trending topic in different scientific fields, such as physics, chemistry and materials science, since Novoselov and Geim reported its successful isolation in 2004 [1]. Its outstanding properties make it an ideal candidate for several applications, such as fabrication of field effect transistors, transparent conductive films, clean energy devices or graphene-polymer nanocomposites with enhanced properties. However, the development of a method for the production of high-quality graphene in large quantities is essential to further exploit its full potential. In this regard, the use of graphene oxide (GO) and reduced graphene oxide (rGO) is a compromise between the interesting properties of graphene, and the synthesis price and complexity. Consequently, GO and rGO can be good substitutes of graphene in many applications.

In particular, graphene-based materials (G, GO and rGO) have been widely used for sensing applications in the last few years due to their high specific surface area, high electronic mobility and low electrical noise. A wide range of chemical sensors, biosensors and gas sensors have been developed using graphene materials [2,3,4,5,6]. 

Among all other sensing strategies, optical fibre sensors have achieved a high impact in the last decades because they offer several advantages over electronic sensors [7,8,9]. One of their main features is that the optical fibre itself can act as both the transmission medium and the transducer, hence allowing remote sensing and multiplexing. Additionally, optical fibre sensors are light and small, resistant to harsh environments and high temperatures, biocompatible, immune to electromagnetic fields and electromagnetically passive [10,11,12]. These features make them particularly suitable for some specific applications, such as biosensing, health and medicine applications, offshore applications and sensing in harsh or flammable environments [8,11,13,14]. 

To date, publications on optical fibre sensors based on graphene materials are limited. However, the unique optical, chemical and morphological properties of graphene combined with the benefits of optical fibre sensing schemes are attracting a growing interest in the scientific community. The increase in the number of publications observed in the last few years is a clear indication of this fact.

This review presents a comprehensive summary of the research on optical fibre sensors based on graphene and its derivatives, including experimental and theoretical studies. The document is structured in the following sections: first, a brief introduction to graphene materials is presented, paying special attention to the different synthesis methods: micromechanical exfoliation, epitaxial growth on SiC substrates, chemical vapour deposition, unzipping of carbon nanotubes, liquid phase exfoliation of graphite, and reduction of exfoliated graphene oxide. The next section is focused on the different optical fibre sensors found in the bibliography, classified by sensing technology (interferometry, surface plasmon resonance, fibre Bragg gratings, absorption and fluorescence). Finally, the conclusions of this review are summarized.

## 2. Graphene Materials

### 2.1. Graphene Discovery

Graphene is the two dimensional form of carbon in which carbon atoms are arranged in a honeycomb crystal lattice such that each atom is joined to three others by sp2-bonding. The use of three σ-electrons in carbon-carbon bonding results in a system of delocalized π-electrons perpendicular to the honeycomb plane giving rise to graphene's exceptional electrical properties [15,16].

The name graphene was introduced by Boehm, Setton and Stumpp in 1986 [17]. For several decades, efforts had been made to produce a single sheet graphene. It was in 2004 that Andre Geim and Konstantin Novoselov reported its successful experimental isolation [1,18]. Authors used a surprisingly simple technique called the “adhesive tape method”. It involved peeling layers of graphite using adhesive tape and then folding and peeling the tape several times to make gradually thinner layers of graphite, ultimately leading to a single layer of carbon. The thinned down graphite was then transferred onto an oxidised silicon substrate and individual small highly oriented pyrolytic graphite domains were identified by means of optical microscopy [1]. 

Since its discovery, graphene has attracted much attention due to its fascinating structural, optical, mechanical and electrical properties [1,6,7,8], which make it an ideal candidate for sensing applications. Additionally, it shows huge potential as a chemical sensing material due to its large surface area [19], sensitivity to changes in the carrier concentration of the transverse Hall resistivity [20], single molecule adsorption detection [21] and ambipolar electric field effect [22], among other properties.

### 2.2. Synthesis Methods of Graphene

**Figure 1:**
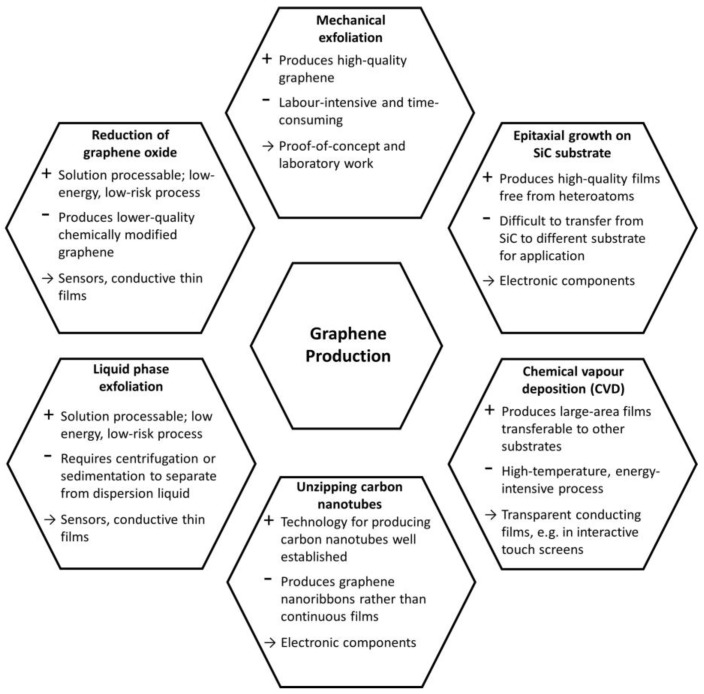
Summary of the main graphene synthesis methods.

Different graphene production processes have been reported, which can be classified in different categories depending on the physical or chemical procedures employed. Figure 1 shows the most common techniques for the production of graphene, which include micromechanical exfoliation (Scotch™ tape method) [1,18], epitaxial growth on SiC substrates [23,24], chemical vapour deposition (CVD) [25,26], unzipping carbon nanotubes [27,28], liquid phase exfoliation of graphite [29,30,31] and thermal & chemical reduction of exfoliated graphene oxide [22,32,33]. Each of these methods has its own advantages as well as limitations depending on its target application.

#### 2.2.1. Micromechanical Exfoliation

As previously mentioned this is an amazingly simple method developed by Novoselov, et al. [1]. Micromechanical exfoliation has high utility for producing single-layer sp2-conjugated domains with high quality structural and electronic properties at up to millimetre size, and is therefore ideal for producing graphene for fundamental physics research and proof-of-concept devices [34]. However, the manual peeling of highly oriented pyrolytic graphite and the subsequent use of microscopy to identify single-layer domains are labour-intensive and time-consuming due to the low throughput of microscopy techniques and the fact that monolayer domains are in the minority among many few- or many-layer platelets [18].

#### 2.2.2. Epitaxial Growth on SiC Substrates

The epitaxial growth of graphene on a SiC substrate involves the fabrication of a graphene film by thermal decomposition on a prepared SiC surface in temperature conditions of up to 1450 °C for up to 20 min [23]. This method is suitable for fabricating graphene-containing electronic components, since SiC is compatible with these applications [35], and produces films that are electrically continuous at a millimetre scale [23]. However, this method of producing graphene has limited applications. The requirement for high temperatures means that this is an energy intensive process. Additionally, it is difficult to transfer graphene epitaxially grown on SiC due to the strong binding between the deposited layer and the substrate [36].

#### 2.2.3. Chemical Vapour Deposition

The chemical vapour deposition (CVD) of graphene films involves the decomposition of a fluid at high temperature to form a film on a substrate. Evaporated Ni film on SiO_2_/Si wafers or copper foils are ideal substrates for graphene synthesis [37,38]. CVD can be used as a relatively high-throughput production method [39] and it has been demonstrated that the deposited graphene film can be transferred from the original substrate to a wide range of other substrates [38,40]. Consequently, this production method is potentially suitable for applications where a graphene film is required on a flexible or polymeric substrate that could not withstand high-temperature processing. The quality of the film deposited onto a substrate depends on the temperature: while achievable at temperatures as low as 300 °C [41], higher temperatures are generally correlated with a more continuous crystalline structure. Therefore, like epitaxial growth the need for high temperatures makes this an energy intensive process.

#### 2.2.4. Unzipping of Carbon Nanotubes

It is possible to ‘unzip’ a one-dimensional carbon nanotube (CNT) (i.e., break a continuous line of bonds along its length or in a helical pattern) to produce a two-dimensional graphene nanoribbon. The procedure for producing CNTs from graphite electrodes using the arc discharge method is well established [42]. Various methods of unzipping single- and multi-walled CNTs (SWCNTs and MWCNTs respectively) have been reported, including: suspension first in concentrated sulphuric acid and then in potassium permanganate in mild conditions [27], argon plasma etching [43], and mechanical sonication in an organic solvent [44]. Although the unzipping of CNTs produces graphene nanoribbons approximately 10–20 nm in width rather than continuous sheets, it is possible to use these ribbons to produce arrays [43].

#### 2.2.5. Liquid Phase Exfoliation of Graphite

The liquid-phase exfoliation of graphite involves the dispersion of graphite flakes in a solvent, ultrasonication of the dispersion to separate individual graphitic layers, and separation of single-layer graphene from remaining multi- and few-layer graphene and from the solvent. This final stage can be achieved using centrifugation or sedimentation. Single-layer graphene can be identified using microscopic and spectroscopic techniques [30,31]. In selecting a solvent, it is necessary to minimise the interfacial tension between the graphite and the liquid in order to minimise the aggregation of single-layer graphene. Surfactants can be used to improve the dispersibility of graphene in the solvent [31], however this may lead to the introduction of heteroatoms to the graphene plane. Although the sonication process tends to produce small flakes with an area of at most 1 μm^2^ [45], these flakes have high utility in solution processing [30].

#### 2.2.6. Reduction of Exfoliated Graphene Oxide

Most of the previously mentioned synthesis methods are unsuitable for commercial-scale graphene production. Fortunately, graphene oxide (GO) is a graphene precursor that can be easily produced at large scale by strong oxidation of graphite using acids via the Hummers’ method [46]. This method enables the exfoliation of GO from bulk graphite at low temperature and in a short period of time. It involves preparing a water-free mixture of powdered graphite, sodium nitrate, sulphuric acid and potassium permanganate, followed by filtration and centrifugation.

GO, an oxidized form of graphene, is decorated by hydroxyl and epoxy functional groups on the hexagonal network of carbon atoms with carbonyl and carboxyl groups at the edges [47,48]. In addition to being easier to produce than pristine graphene, the oxygen-containing functional groups of GO give hydrophilicity, which can be very important for the large-scale uses of graphene as it enables its dispersion into some solvents for film deposition [49]. However, the presence of oxygenated functionalities in GO significantly diminishes its electrical conductivity compared to pristine graphene due to the disruption to the conjugated π-electron system [50]. Consequently, for some applications it is essential to remove some oxygen-containing functional groups by means of reduction, in order to partially restore the valuable properties of graphene. The material derived from the reduction of GO is called partially reduced graphene oxide (rGO) or chemically converted graphene (CCG). Several reduction methods have been reported [32,49,51,52,53,54]. They result in different properties of the obtained rGO. The reduction rate will influence the performance of the final application. 

• Thermal reduction of exfoliated graphene oxide

The thermal reduction is believed to be a green method because no hazardous reductants are used. GO can be reduced by thermal treatment and the process is named thermal annealing reduction. The key parameters in this reduction method are the annealing temperature and the annealing atmosphere. This process requires heating up to 1000 °C under vacuum [55] or inert atmosphere [56]. Nevertheless, in hydrogen atmosphere the reduction can be carried out at much lower temperatures due to its high reduction ability [57,58].

Thermal annealing is an effective reduction method, however, due to the temperatures required, it is very energy intensive. In addition, some applications need the deposition of a GO thin film on a specific substrate, such as polymers, therefore this approach cannot be used to reduce GO films deposited on substrates with low melting-points.

• Chemical reduction of exfoliated graphene oxide

Chemical reduction of GO involves its reaction with different chemical reducing agents. Hydrazine and its derivatives (hydrazine hydrate and dimethylhydrazine) have been accepted as the best reducing agents [21,22,33,49]. The GO reduction is achieved by the addition of the reducing agent to the GO dispersion, obtaining agglomerated graphene-based nanosheets due to the increased hydrophobicity.

A significant issue is the dangerousness of these reductants, being toxic, hazardous, explosive and not environmentally benign. Consequently, continuous research has been focused on the development and optimization of eco-friendly reducing agents for GO reduction. Electron transfer reactions have been demonstrated to partially reduce graphene oxide in reactions involving alcohols [59], vitamin C [60], and in high-pH solvents [61].

• Other reduction methods of exfoliated graphene oxide

A diverse range of alternative methods for the reduction of exfoliated graphene oxide have been proposed, including electrochemical and photolysis-based processes. The examples given are low-temperature methods with minimal heteroatom contamination of the reduced graphene oxide. 

The photolysis of graphene oxide by UV light, resulting in an order of magnitude improvement in conductivity, has been demonstrated to proceed quickly when catalysed by TiO_2_ or ZnO [62]. It has also been shown that UV photolysis can be used for the partial reduction of isolated solid graphene oxide [63,64] and for graphene oxide in an aqueous suspension [65]. By contrast, the photolysis of graphene oxide films using lasers has been demonstrated using the relatively simple technique of depositing a graphene oxide film onto a DVD and using the laser of a DVD drive to produce a freestanding film with high conductivity (1738 Sm^−1^) [66].

Numerous methods have been developed to synthesise graphene, however high yield and cost-effective production of defect-free graphene at large-scale is not widely available, which is crucial for real-world applications. The use of GO and rGO achieves a compromise between partial recovery of the conjugated electron system, high scalability of production and suitability for solution processing, making them ideal candidates for commercial applications. Consequently, research efforts in the field of optical fibre sensors have mainly focused on the use of GO and rGO as sensing coatings.

## 3. Optical Fibre Sensors Using Graphene-Based Materials

### 3.1. Interferometry Based Optical Fibre Sensors Using Graphene-Based Coatings

Optical fibre interferometers use the interference between two beams that propagate through different optical paths (of a single fibre or different fibres). If one of the optical paths is affected by external perturbations, the interference will be also affected. Interferometric signals give huge temporal and spectral information. For this reason, the measurand can be quantitatively determined through different properties of the optical signal such as wavelength, phase, intensity, frequency or bandwidth [67]. There are two main groups of optical fibre interferometers: Fabry-Perot (FPI) and Mach-Zehnder (MZI).

**Figure 2:**
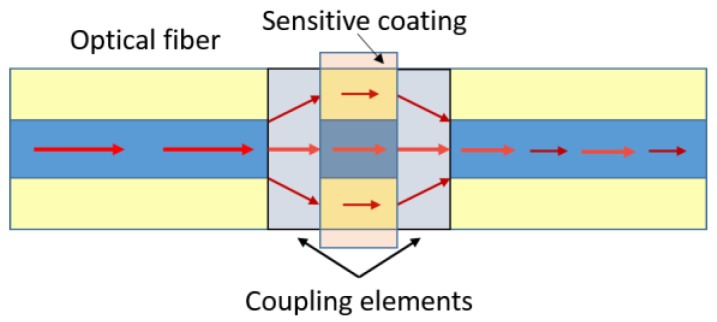
Schematic representation of a MZI-optical fibre sensor.

MZIs have been widely used for optical fibre sensing applications due to their flexible configurations. The early MZIs had two independent arms, the reference arm (isolated from external variations) and the sensing arm (exposed to the variations of the external medium). An incident light is split into both arms by a fibre coupler and then recombined by another fibre coupler to obtain the interference signal. The two-arm scheme was replaced by a more versatile in-line scheme. In this new generation of optical fibre MZIs a part of the beam guided through the core of an optical fibre is coupled to cladding modes of the same fibre by an intercalated element, and then re-coupled to the core mode by another intercalated element. In these in-line MZIs both the reference arm and the sensing arm have the same physical length. However, as the cladding mode beam has a lower effective refractive index than the core mode beam they have different optical lengths due to the modal dispersion [67]. Different configurations of MZIs can be found depending on the coupling strategy used, such as long period gratings, photonic crystal fibres, core mismatch, fibre tapering, etc. In Figure 2 schematic representation of an optical fibre MZI can be found.

Some authors have used graphene-based materials in MZI configurations to obtain optical fibre sensors. Yao et al. [68], report an ammonia sensor based on a graphene/microfibre hybrid waveguide. The sensing mechanism relies on the modification of the graphene conductivity because of the adsorption of ammonia. Consequently, the effective refractive index of the device and the light transmitted along it are very sensitive to ammonia concentration. A sensitivity of ~6 pm/ppm was obtained using this approach. 

**Figure 3:**
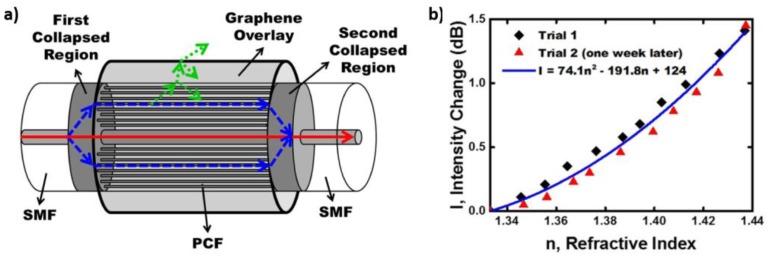
(**a**) Schematic representation of the refractive index sensing element formed by the deposition of a graphene overlay onto an MZI; (**b**) Change in intensity of the interference vs. RI for two separate trials (diamonds and triangles) conducted one week apart. Reprinted with permission from [69].

Tan et al. presented a refractometer that involved the deposition of a graphene overlay onto the surface of a photonic crystal fibre (PCF) segment in a fibre-based MZI (Figure 3) [69]. This sensor achieved a sensitivity of 9.4 dB/RIU for RIs between 1.33 and 1.38 and a sensitivity of 17.5 dB/RIU for RIs between 1.38 and 1.43.

A similar device using GO as sensitive coating has been recently reported by Dash et al. [70]. When the RI of the analyte is changed from 1.3310 to 1.3715, the wavelength of the dip shifts from 1544.4 nm to 1553 nm (wavelength sensitivity of 212 nm/RIU) and the intensity of the dip also changes from −78.16 to −83.43 dBm (intensity sensitivity of 130 dB/RIU). This sensitivity is higher than the previously reported results based on similar configurations without any coating [71,72].

**Figure 4:**
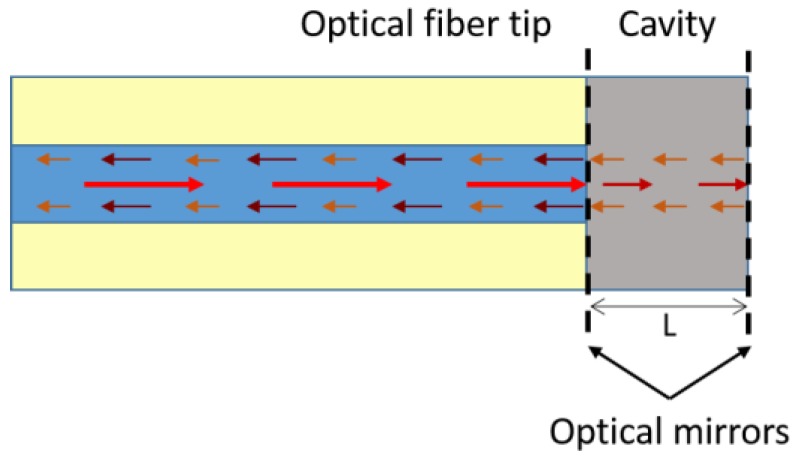
Schematic representation of an extrinsic Fabry-Perot interferometer on the tip of an optical fibre.

An FPI consists of two parallel optical mirrors separated by a certain distance. Interference occurs due to the multiple additions of reflected and transmitted beams at the two mirrors. In the case of optical fibres, FPI sensors can be classified into extrinsic and intrinsic. Extrinsic FPIs use the reflections from an external cavity formed outside the fibre, as shown in Figure 4. This cavity can be built using an air space and a diaphragm or a coating made of a sensitive material. Intrinsic FPIs sensors have reflecting components within the fibre itself [67].

**Figure 5:**
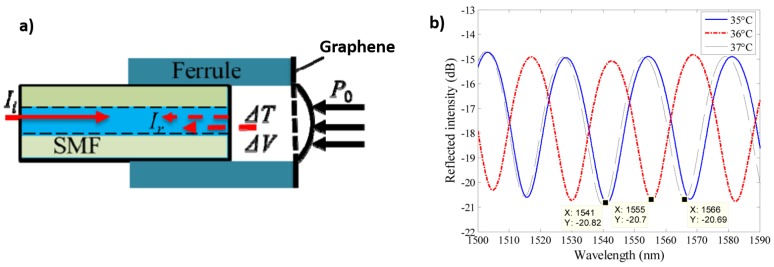
(**a**) Schematic diagram of the FP sensor; (**b**) Reflection spectra of the FP sensor for different temperatures. Reprinted with permission from [73].

Li and co-workers from Beihang University (China) have developed in the last few years a wide variety of sensors based on FPI using a G diaphragm. Figure 5a shows the schematic diagram of these FPI sensors which include a zirconia ferrule, a standard single mode fibre (SMF) and a multi-layer graphene diaphragm. The diaphragm, adhered to the zirconia substrate by van der Waals forces, acts as a light reflector. When this device was tested as a temperature sensor [73], the variation of the cavity length was approximately 352 nm/°C in the temperature range from 20 °C to 60 °C. This effect is induced by the thermal deformation of the graphene diaphragm. However, the intensity and phase shifts at common temperatures featured a periodic appearance even due to a narrow thermal fluctuation (Figure 5b). This group has used similar devices to develop sensors for pressure [74], adhesion energy [75] and thermal expansion coefficient [76] of graphene. A similar approach was used in [77] to obtain an optical fibre acetylene detector with low level detection of acetylene and a lower detection limit of 119.8 ppb.

### 3.2. Surface Plasmon Resonance Optical Fibre Sensors Using Graphene-Based Coatings

Over the past two decades, surface plasmon resonance (SPR) based sensors have attracted the attention of many researchers due to their potential applications in the field of physical, chemical and biomedical sciences [78,79,80,81,82,83,84]. A surface plasmon is a transverse magnetic (TM) polarized electromagnetic wave excited by p-polarized light. Due to the exponential decay of the plasmon electric field, it is strongly localized at the metal-dielectric interface. When a plasmon is excited, an absorption peak (SPR) at a determined wavelength (resonance wavelength, λ_SPR_) is produced [79].

**Figure 6:**
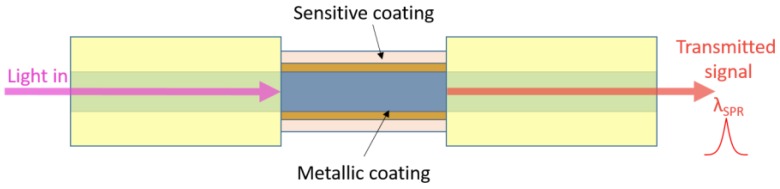
Schematic representation of a SPR-based optical fibre sensor.

In the case of optical fibre-based SPR sensors, the device shown in Figure 6 is typically used to excite a surface plasmon. The cladding from a small portion of the fibre is removed and this unclad portion is coated with a thin layer of metal. The light from a polychromatic source is coupled into the fibre from one end and the spectrum of the transmitted power at the other end is collected. Due to the SPR, a peak at λ_SPR_ is obtained in the transmitted spectrum. This λ_SPR_ shows a strong dependence on the refractive index of the sensing medium around the metal layer. Using this scheme, a great variety of sensors can be obtained just by depositing onto the metal thin-film a material that is sensitive to the chemical compound or physical property of interest [85,86].

In the last few years, some studies that include graphene materials in optical fibre SPR-based sensors have been published. In these studies, graphene materials can play different roles. Some authors have used them as SPR supporting materials instead of the typically used gold and silver layers or in addition to them. In other cases, the authors have utilized graphene-based coatings as the sensitive material, which reacts to any variation of the target analyte. In the next paragraphs, some examples of these sensors are introduced.

Kim et al. used graphene in a SPR sensor as replacement material for gold or silver [87,88]. A multi-layered graphene film was synthesized by (CVD) on a Ni substrate and transferred on the sensing region of an optical fibre. The graphene coated SPR sensor showed a good sensitivity when used to analyse the interaction between structured DNA biotin and Streptavidin.

In [89], the authors presented a theoretical study of an SPR biosensor for detection of bonding between adenine and thymine or between guanine and cytosine (DNA hybridization). They selected gold as SPR-generating layer and introduced a multilayer graphene structure on its surface. The proposed sensor seemed to be more sensitive than conventional biosensors without the graphene layer. Additionally, the sensitivity linearly increased with the increase in the number of graphene layers. In particular, an improvement of 25% in the sensitivity was achieved by adding 10 G layers to the conventional gold thin film SPR biosensor. This improvement is mainly due to the better adsorption of DNA molecules on G than on gold. Fu et al. [90] proposed a similar approach to demonstrate theoretically the enhancement in sensitivity of a SPR refractive index sensor with G layers onto a gold SPR-supporting layer. 

**Figure 7:**
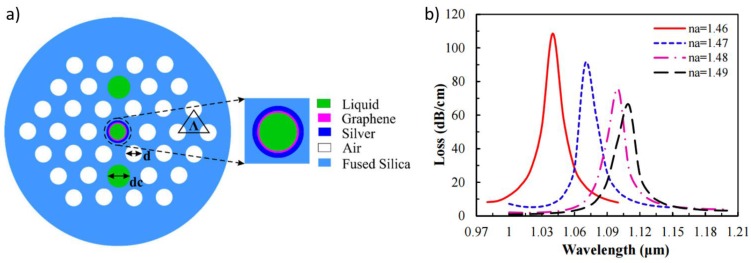
(**a**) Cross-section of the SPR-based sensor proposed in [91]. (**b**) Loss spectrum of the fundamental mode by increasing analyte RI from 1.46 to 1.49. Published under a Creative Commons Attribution License (CC-BY).

In [91], the authors simulated the performance of a photonic crystal fibre (PCF) SPR-based refractive index sensor. A silver layer deposited onto the inner surface of one of the PCF holes acted as SPR supporting layer and a G layer deposited onto the silver coating was used to inhibit its oxidation (see sensor cross section in Figure 7a). The analyte, a liquid with high RI, was infiltrated into the deposited channel hole and the fibre cores. The proposed sensor showed a maximum RI sensitivity of 3000 nm/RIU and an average RI sensitivity of 2390 nm/RIU in the sensing range of 1.46 to 1.49 (see Figure 7b). This sensitivity is slightly lower than the achieved by other works using a similar structure with a single gold layer [92].

Mishra and co-authors carried out a detailed study about the fabrication and characterization of SPR-based fibre optic gas sensors using rGO, carbon nanotubes (CNTs) and poly(methyl methacrylate) (PMMA) [93]. Probes with a silver SPR-supporting layer were coated with different sensitive materials (rGO, CNTs, rGO-CNTs and rGO/CNT/PMMA hybrid nanocomposite) in order to achieve the best performance sensor for the detection of several gases (methane, ammonia, hydrogen sulphide, chlorine, carbon dioxide, hydrogen, and nitrogen). Among all the tested possibilities, the sensor based on rGO/CNT/PMMA hybrid nanocomposite showed a high selectivity to methane. The sensitivity of this device was optimized by varying the concentration of rGO/CNT in PMMA. The optimum doping concentration was 5 wt. % and the maximum sensitivity was 0.33 nm/ppm in the range for methane gas concentrations from 10 to 100 ppm. The same group developed an ammonia sensor based on SPR using a copper supporting layer and rGO/PMMA hybrid composite, obtaining a maximum sensitivity close to 1 nm/ppm in the same range of ammonia concentration [94].

A particular case of SPR is localized surface plasmon resonance (LSPR). When a very thin dielectric coating that includes metal nanoparticles (typically gold or silver) is deposited onto a waveguide, a resonant coupling between the incident electromagnetic wave and the surface of the thin-film is produced. This results in an electromagnetic wave at the metal-dielectric interface that causes appearance of a sharp absorption peak at a determined wavelength in the transmitted spectrum [95,96].

Nayak et al. developed different refractive index sensors using graphene oxide encapsulated gold nanoparticles (GOE-AuNPs) [97] and graphene oxide-encapsulated silver nanoparticles (GOE-AgNPs) [98] as LSPR generating materials. The main benefits of encapsulating the nanoparticles in GO are the control of the inter-particle distance, preventing aggregation, the enhancement of the colloidal stability and the prevention of the oxidation of the AgNPs, avoiding their direct contact with the aqueous medium. The variation in the absorbance of the LSPR peak when the device was immersed in aqueous solutions with refractive indices between 1.34 and 1.38 produced sensitivities of 2.288 ΔA/RIU and 0.9406 ΔA/RIU for GOE-Au NPs and GOE-AgNPs, respectively.

### 3.3. Fibre Bragg Gratings Sensors Using Graphene-Based Coatings

**Figure 8:**
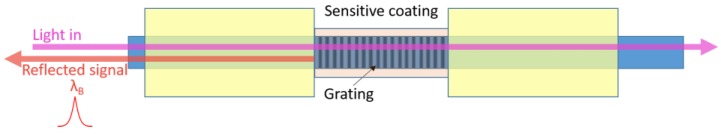
Schematic representation of an eFBG coated with a sensitive thin film.

Fibre gratings consist of a periodic perturbation of the properties of the optical fibre, generally of the refractive index of the core (Figure 8). In Fibre Bragg gratings (FBGs), this alteration produces a coupling of light from the forward-propagating mode of the optical fibre to a backward propagating mode. This coupling occurs at a specific wavelength that depends on the period of the FBG and the effective index of the propagating mode. As a consequence, a variation in either of these parameters produce a change in the coupling wavelength (Bragg wavelength, λ_B_) that can be measured. As strain and temperature have a direct influence on the mentioned parameters, many sensing schemes based on FBGs have been developed to measure these signals [99,100,101,102,103,104,105,106,107].

FBGs can be also used for chemical sensing and biosensing. For this purpose, FBGs are coated with a material whose structure varies in the presence of a particular chemical compound (analyte). When the concentration of this analyte changes, the deformation of the coating produces an axial strain. The FBG stretches or shrinks under such strain and therefore the spectral pattern of reflected light changes, producing a shift in reflected wavelength [108]. If the fibre core of the FBGs is covered by the cladding layer, it is less sensitive to the variations in the surrounding medium. To overcome this issue, the cladding is etched in order to expose the propagating modes of the core to the surrounding medium [109]. A schematic representation of an etched FBG (eFBG) coated with a sensitive thin-film is shown in Figure 8. These devices have been used for chemical applications such as refractive index sensing [106], gas sensors [108] or relative humidity sensors [110] and also for biosensing applications [109,111,112].

In the last few years, an increasing number of studies about optical fibre sensors based on FBGs have been focused on devices that include coatings made of graphene-based materials. In this section, different examples of these structures are presented.

The Sood group from the Indian Institute of Science (Bangalore, India) has intensively studied this type of sensor. They have developed different sensing schemes based on etched FBGs including gas detectors, physical sensors and biosensors. In particular, they have enhanced the typical sensitivity of bare FBGs to strain and temperature by coating with rGO an etched FBG. These sensors showed a sensitivity to strain of 5.5 pm/με and a sensitivity to temperature of 33 pm/°C (5 and 3 times better than bare FBGs). The resolutions obtained with these sensors were about 1 με for strain measurements and 0.3 °C for temperature measurements [113].

Furthermore, the same group has developed different biosensors based on this configuration. In [109], they presented an etched FBG coated with aminophenylboronic acid (APBA)-functionalized rGO that exhibited high sensitivity to glucose. These sensors showed a linear shift in Bragg wavelength with the concentration of a glucose solution in the range of 1 nM to 10 mM, covering the clinical range of the estimated average glucose concentration in red blood cells, which enables them to be used in detection of diabetes. They have also designed and characterized biosensors for proteins CRP [111] and concanavalin A [114] detection using this sensing scheme.

Other research groups have also exploited the mentioned mechanism. Wang et al. have recently developed a relative humidity sensor consisting of a tilted FBG coated with a GO thin film obtaining a maximum sensitivity of 0.129 dB/%RH in the relative humidity range from 10% to 80% [115]. 

**Figure 9:**
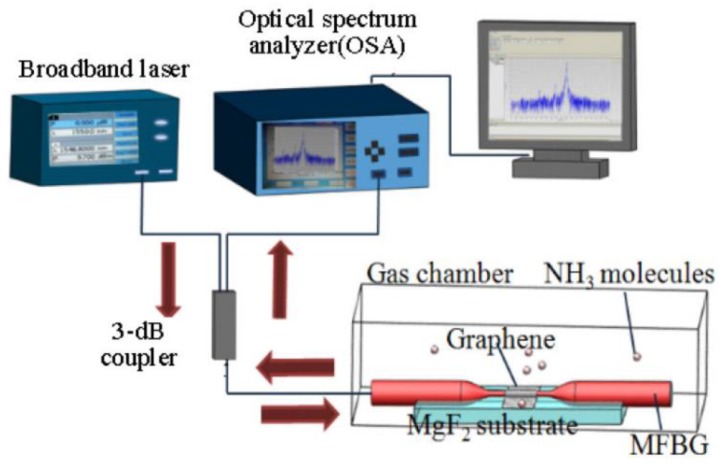
Schematic representation of the experimental setup used in the characterization of an ammonia sensor consisting of a graphene-coated eFBG. Reprinted with permission from [116].

**Figure 10:**
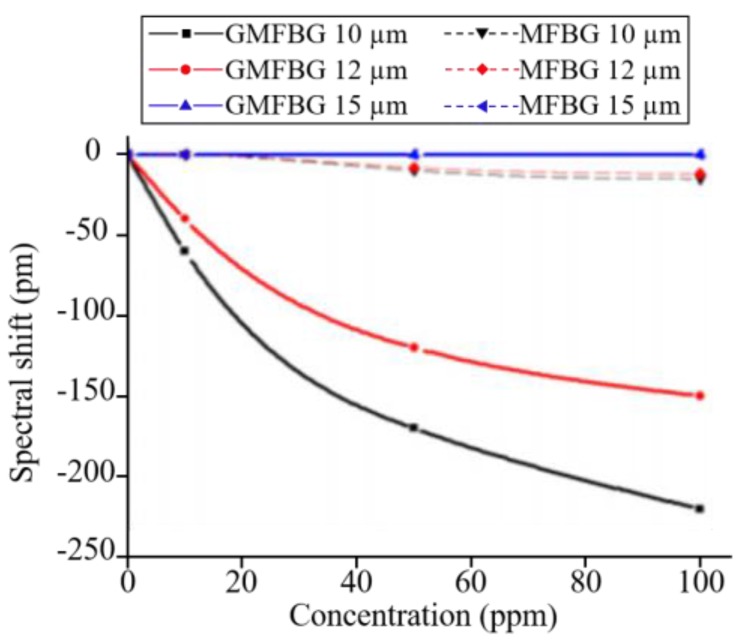
Spectral shifts of the GMFBGs and MFBGs for different concentrations of NH_3_. Reprinted with permission from [116].

Zhang et al. studied the features of a graphene-coated microfibre FBG (GMFBG) as an ammonia sensor. Figure 9 shows the experimental setup used in this study [116]. Different diameters of GMFBGs were tested for NH_3_ gas with the concentrations of 0 ppm, ~10 ppm, ~50 ppm, and ~100 ppm. To further demonstrate the enhancement effect by graphene, bare microfibre FBGs (MFBGs) with different diameters were also tested. The results of these experiments are shown in Figure 10. It can be concluded that MFBGs without the graphene cladding were almost not sensitive to gas adsorption while GMFBGs showed a maximum sensitivity of 6 pm/ppm for 10 µm diameter microfibres. These results also indicate that the GMFBG with a smaller diameter were more sensitive to the gas concentration alteration.

### 3.4. Absorption-Based Optical Fibre Sensors Using Graphene-Based Coatings

FBGs/LPGs and optical fibre interferometers require complex fibre optic pre-processing or sophisticated control at the micrometre level respectively. In contrast, optical fibre sensors based on light intensity monitoring, such as transmission, absorption or reflection, are relatively simple to implement. On the other hand, these systems are vulnerable to light intensity variations, light source instabilities, micro and macro bending or external source coupling. In order to overcome these undesired effects these systems use complex detection algorithms and systems with referenced measurements, which are not associated with the light intensity variations produced by the selected analyte [117,118].

Absorption-based optical fibre sensors rely on the fact that the selected target or transducer must modify the intensity of the light propagated through the optical fibre core as a function of the selected measurand. Here, the intensity loss can be described using the Beer-Lambert law:

I_1_ = I_0_e^−αl^ (1)

where l is the length of the fibre sensitive region, I_0_ and I_1_ are the excitation and transmitted light intensities and α, the absorption coefficient, can be expressed as α = Cε/loge where C is the molar concentration and ε is the molar absorptivity. The absorbance (A), is proportional to the length of the fibre sensitive region and the analyte concentration and can be obtained from Equation (1) and expressed as:

A = log(I_1_/I_0_) = εCl (2)

**Figure 11:**
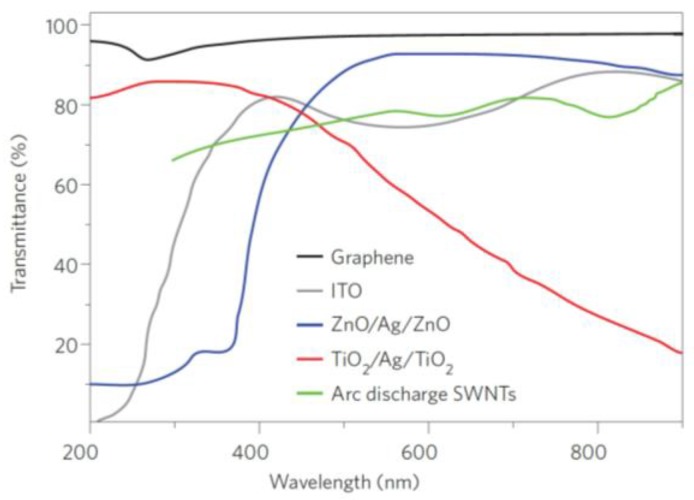
Transmittance of different transparent conductors: Graphene, indium tin oxide (ITO), ZnO/Ag/ZnO, TiO_2_/Ag/TiO_2_ and arc discharge single-walled nanotubes. Reprinted from [119] with permission from Nature Photonics.

Optical fibre geometry is generally modified in this type of sensor in order to enhance light interactions with the selected measurand or transducer. Some typical examples are the cases of U-bent, side polished, cladding removed, microstructured, tapered fibres and so on. Concerning these intensity-based measurements, the general idea is based on the fact that the optical properties of the target or transducer are altered as a function of the selected magnitude or analyte concentration. More specifically, the optical absorption of graphene layers can be considered proportional to the number of layers with little or no perturbation between adjacent layers. Graphene also exhibits a quite flat response from 300 to 2500 nm with an absorption peak near 270 nm as shown in Figure 11 when it is compared with other transparent conductors.

The two-dimensional hexagonal shape structure of graphene, large surface area and high electron mobility enable it to adsorb easily different kinds of gas molecules, volatile organic compounds (VOCs) and biological species [120]. Molecule adsorption on graphene’s surface can modify the electrical conductivity and alter the complex refractive index value, which results in variations of the absorption spectrum. Thus, the detection and concentration measurement of different compounds is performed by simply measuring the absorption spectrum [121].

The next paragraphs will focus the attention on the utilization of thin graphene-based coatings, fabricated onto diverse optical fibre sensing schemes, as transducers for the detection of physical magnitude or property changes such as temperature or UV radiation as well as chemical and biological compounds: relative humidity, ethanol, ammonia, glucose or DNA. These applications, together with the sensing characteristics of the obtained devices are also summarized in Table 1.

Graphene-based thin-films can be exploited for the fabrication of optical fibre sensors. Ambient temperature, humidity or ultra violet light radiation among others have a great impact on the conductivity of graphene, leading to variations of the effective refractive index and having a measurable effect on the transmitted optical power. Fast response all-fibre graphene assisted temperature sensors have been obtained using microfibre [122] and side-polished [123] optical fibre structures with sensitivities of 0.134 dB/°C and 0.1018 dB/°C respectively for a wide range of temperatures. The fabrication of UV light exposure sensors has been explored by means of the utilization of highly birefringent fibre covered with graphene oxide (GO) [124] and tapered SMF microfibres in contact with methylene-blue functionalized reduced graphene oxide (MB-rGO) [125]. Light intensity modulation of rGO at high relativity humidity range (70%–95%) was also studied in [126] using side-polished single mode fibres (SMF) obtaining a sensitivity of 0.31 dB/%RH and a response time faster than 0.13% RH/s.

**Figure 12:**
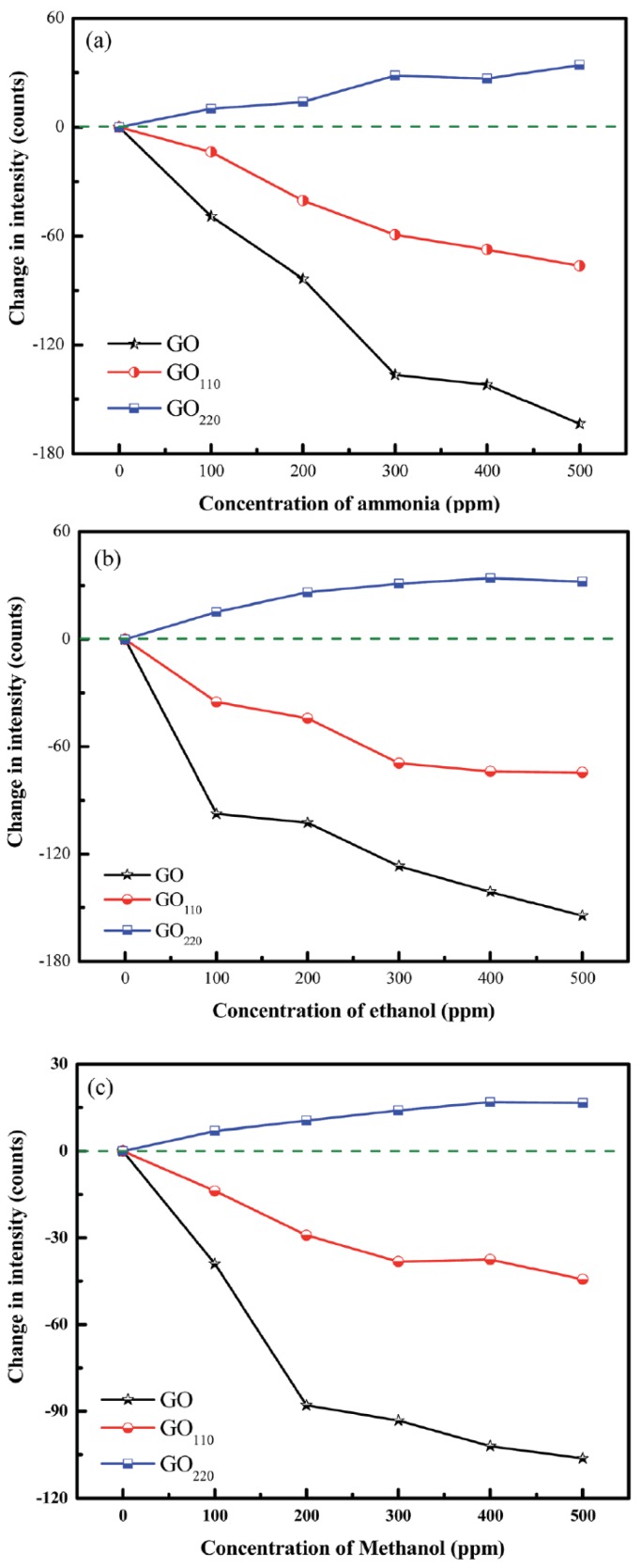
Change in output intensity at gas concentrations between 0 and 500 ppm of (**a**) ammonia (**b**) ethanol and (**c**) methanol for GO and rGO (GO_110_ and GO_220_). Reprinted from [132].

Carbonyl and carboxylic acid functional groups present in GO show better affinity than graphene to capture ethanol or benzene molecules in aqueous solutions. Several authors have explored this advantage in order to develop ethanol sensors using diverse optical fibre architectures, such as tapered multimode fibres (MMF) [127,128,129,130], or U-bent fibres [131]. The combination of high surface area with the hydrophilic and hydrophobic properties of GO and rGO respectively is also very interesting for the detection of gaseous species and VOCs. In particular, Kavinkumar et al. [132] studied the role of functional groups in the absorption properties of cladding-removed multimode fibres (CRMMF) coated with GO and rGO (heated at 110 °C, GO_110_, and heated at 220 °C, GO_220_) when exposed to gaseous ammonia, ethanol and methanol (see Figure 12). These results evidence good device sensitivity, but minimal selectivity in the response.

**Figure 13:**
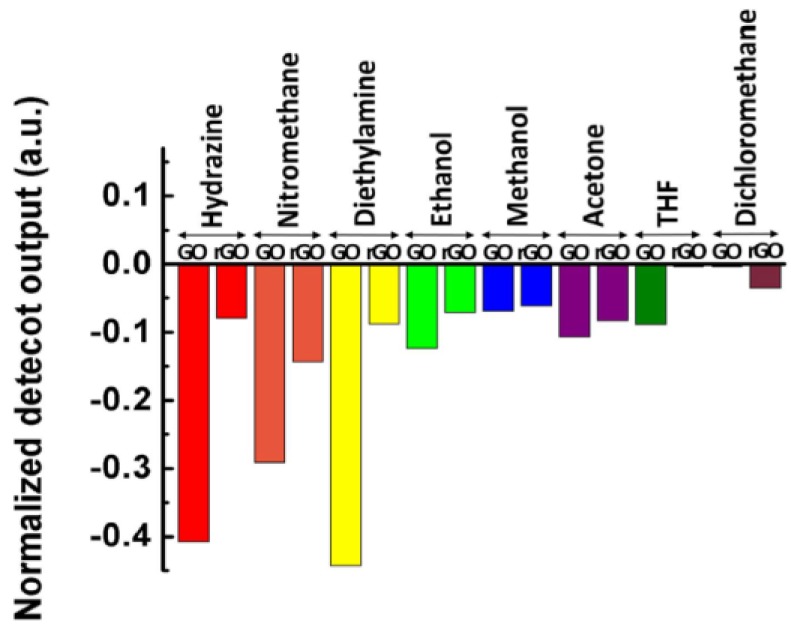
Comparative plots of the sensing responses of GO and rGO to eight different vapours at a 500 ppb concentration level. Reprinted from [133].

High to sensitive and selective VOCs detection has been also explored in [133] by Some and co-workers by means of the fabrication of GO, rGO and mixed GO/rGO thin-films onto the end tip of plastic optical fibres (POF) as it is represented in Figure 13. 

Some other works have explored the affinity of graphene and graphene-based materials for the detection of more complex molecules. For example, Qiu and co-workers explored glucose and double strand DNA (ds-DNA) detection by means of the fabrication of a single-layer graphene onto tapered POF (TPOF) [134] and tapered MMF (TMMF) [135] respectively. However, further research is required in this field in order to establish a good linear sensitivity relationship and guarantee the selectivity of the fabricated devices with the selected analyte.

### 3.5. Fluorescence-Based Optical Fibre Sensors Using Graphene-Based Coatings

Fluorescence or phosphorescence measuring systems, referred to as fluorescence henceforward, have been established as an important group of optical fibre sensors. This technique has been traditionally used in analytical chemistry or biochemical applications due to its excellent performance, including time saving, fast response, cost effectiveness, high sensitivity and specificity and good reproducibility. The detection mechanisms are based on fluorescence lifetime or intensity measurements using modulated, pulsed or continuous excitation light sources [136]. The fluorescence of the active material can be enhanced or quenched as a function of the presence and concentration of the target molecule. Concerning the fluorescence intensity, it is important to take into account the excitation intensity and the efficiency of the active material, which is the ratio between the emitted and absorbed photons in the active material. In the case of optical fibre fluorescence-based sensors, where the optical fibre is also used to transmit the fluorescence signal to the detector, it is also important to consider an optimal design to couple the maximum fluorescence emission into the fibre, such as in the case of microstructured optical fibres [137]. Moreover, photodegradation of the active material, photobleaching after long time exposure to the excitation source or self-quenching at high target concentrations are some of the drawbacks of these devices. Thus, as in the case of absorption-based sensors, a referenced signal is required in order to avoid undesired power fluctuation effects [138].

**Figure 14:**
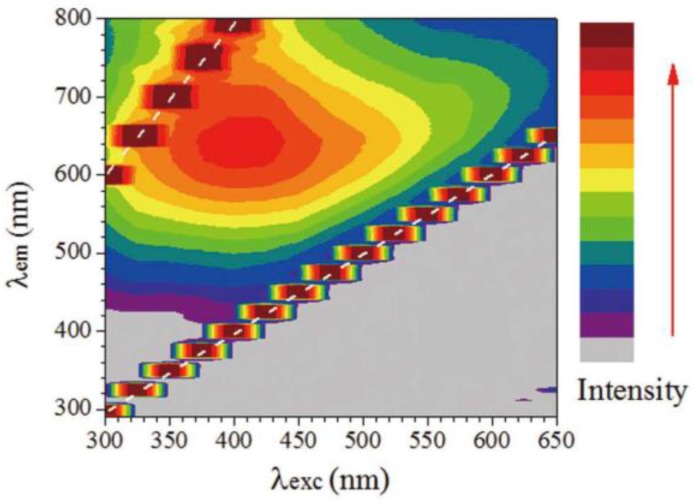
Fluorescence intensity excitation-emission map of GO; strong signals indicated by dashed white lines are due to scattering of excitation light and its second order. Reprinted from [139].

GO is fluorescent over a broad range of wavelengths, owing to its heterogeneous electronic structure. Blue to green fluorescence emission can be obtained as a function of the light excitation wavelength with a redshift of the fluorescence maximum intensity with the increase of excitation wavelength above 400 nm as shown in Figure 14.

In particular, GO fluorescence can be obtained by inducing a bandgap using two preferred routes. One consists of producing graphene ribbons and graphene quantum dots (GQDs) while the other is based on chemical and physical treatments, such as oxygen plasma treatments [119,140,141]. While intrinsic GO fluorescence is interesting, much more needs to be understood about its properties in relation to quenching by external (analyte) mediators, measurement conditions etc. before this property can be fully exploited for sensor development.

**Figure 15:**
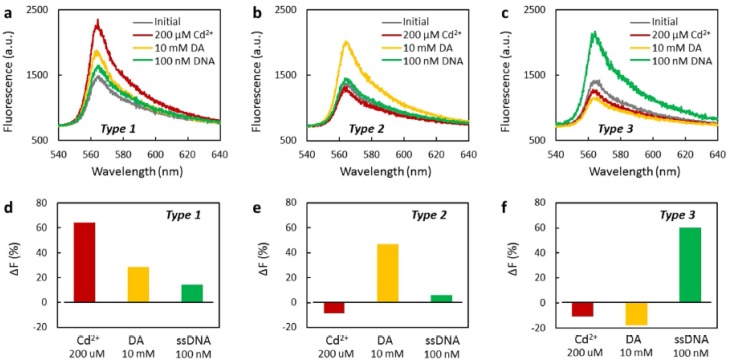
Fluorescence spectra of Type 1 (**a**), Type 2 (**b**) and Type 3 (**c**) sensor when immersed in 200 μM Cd^2+^ (red curve), 10 mM DA (yellow curve) and 100 nM (green curve).(**d**–**f**), Histograms: the fluorescent restoration ratio of Type 1 (**d**), Type 2 (**e**) and Type 3. Reprinted from [142] under a Creative Commons CC-BY license.

Partially rGO (prGO) with adsorbed fluorescent rhodamine 6G (Rh6G) molecules (Type 1) was coated onto etched MMF for the detection of Cd^2+^ ions, dopamine (DA) and single-strand DNA (ssDNA) [142]. Additional processing treatments were required to enhance the selectivity of the prGO with adsorbed Rh6G molecules to the specific analyte, such as nitrate pre-immersion in Type 2 sensor (to make the device not absorbable to ions and immune to DA agglomeration), or Na^+^ functionalization in Type 3 sensor (to form COO^-^Na^+^ bindings). Fluorescence measurement results from the obtained devices are shown in Figure 15. In particular, lower detection limits of 1.2 nM, 1.3 μM and 1 pM were achieved for cadmium ion, DA and ssDNA respectively.

## 4. Conclusions and Future Trends

The exceptional electrical, mechanical, thermal and chemical properties of graphene offer an ample range of possibilities for the development of optical fibre sensors based on different interrogation schemes, such as resonance, interferometry, fibre Bragg gratings, light intensity modulation or fluorescence. Some application examples for temperature, humidity, UV light and VOCs sensing have been reviewed in the above sections.

The use of graphene and graphene-based materials for sensing applications is at a very early stage. It still needs some time and effort to achieve its full potential. In particular, the understanding of the surface chemistry of GO and rGO, the role of functional groups, the modification of graphene structure by means of the introduction of defects and dopants could all contribute to new approaches to enhance the sensitivity and selectivity to gaseous compounds and more complex molecules. These advances combined with novel micro and nanofabrication techniques would enable the fabrication of 3D graphene structures at the nanoscale level opening the door to novel applications. It is also important to improve coating strategies and in-situ reduction of GO to enhance the homogeneity and reproducibility of films. GQDs, which consist of single atom graphene sheets with a thickness in the order of 3~20 nm, have emerged as a good alternative for applications that require high fluorescent activity, robust chemical inertness, long fluorescence lifetime and excellent photostability when compared with other carbon-based nanomaterials [128]. In addition, the excitation and pH dependent fluorescence wavelength emission of GQDs has attracted enormous interest within the scientific community linked to the understanding of those emissions as well as to the potential applications in photovoltaic devices, biosensing and imaging, which foresees a promising future of graphene-based materials in this area.

## Figures and Tables

**Table 1:** Summary of graphene-based optical fibre sensors.

Detection Mechanism	Material	Optical Fibre Configuration	Analyte	Dynamic Range	Sensitivity	Response Time	Ref.
MZI	GO	PCF MZI	RI	1.33–1.37	212 nm/RIU 130 dB/RIU	-	[70]
MZI	G	PCF MZI	RI	1.38–1.43	17.5 dB/RIU	-	[69]
MZI	G	2-arms MZI	Ammonia	40–360 ppm	~6 pm/ppm	0.4 s	[68]
SPR	Cu + rGO/PMMA	Uncladded MMF	Ammonia	10–100 ppm	~1 nm/ppm	-	[94]
SPR	Ag + rGO/CNT/PMMA	Uncladded MMF	Methane	10–100 ppm	0.33 nm/ppm	-	[93]
LSPR	GOE-Au NPs	Uncladded MMF	RI	1.34–1.38	2.288 ΔA/RIU	-	[97]
LSPR	GOE-Ag NPs	Uncladded MMF	RI	1.34–1.38	0.940 ΔA/RIU	-	[98]
Bragg wavelength	rGO	Etched FBG	Strain and Temperature	-	5.5 pm/μє 33 pm/°C	-	[113]
Bragg wavelength	rGO	Etched FBG	NO_2_	0.5–3 ppm	-	6 to 20 min	[143]
Bragg wavelength	APBA-rGO	Etched FBG	Glucose	1 nM–10 mM			[109]
Bragg wavelength	Dendrimers-GO	Etched FBG	Lectin Con A	500 pM	-	-	[114]
Bragg wavelength	antiCRP-GO	Etched FBG	CR Protein	0.01–100 mg/L	6.3 pm/CRP magnitude order	-	[111]
Bragg wavelength	p-doped G	D-shaped polymer FBG	Erythrocyte	-	1 pm/ppm	-	[144]
Bragg power at 1557 nm	GO	Tilted FBG	Relative Humidity	10%–80%HR	0.129 dB/%RH	-	[115]
Bragg wavelength	PMMA-G	MFBG	Ammonia	0–100 ppm	6 pm/ppm	-	[116]
Absorption	GO	Tapered MMF	Aqueous Ethanol	-	-	20–30 s	[129]
Reflectance	GO	Tapered MMF	Aqueous Ethanol	5%–80%	0.02–0.0275 ∆R/∆C ^1^	19–25 s	[130]
Absorption	GO	Tapered MMF	Aqueous Ethanol	5%–40%	0.829 ∆A/∆C ^1^	15–40 s	[127,128]
Absorption	GO	U-bent MMF	Aqueous Ethanol	5%–100%	0.44–0.0925 ∆A/∆C ^1^	1–2 s	[131]
Absorption	GO, rGO	CRMMF	Ethanol, methanol, ammonia	0–500 ppm	0.26, 0.2 and 0.32 counts/ppm	-	[132]
Absorption	Graphene/PANI	Side-polished MMPF	ammonia	0%–1% vol.	4.1 ∆A/∆vol. ^1^	24–71.8 s	[145]
Absorption	GO-rGO	POF	VOCs	-	-	-	[133]
Absorption	rGO	Side-polished SMF	Temperature	−7.8–77 °C	0.134 dB·°C^−1^	-	[123]
Absorption	rGO	Tapered SMF	Temperature	30–80 °C	0.1018 dB·°C^−1^	-	[122]
Absorption	rGO	Side-polished SMF	Humidity	70%–95% RH	0.31 dB/%RH	0.13% RH/s	[126]
Absorption	GO	Tapered POF	Glucose	1%–40% vol.	-	-	[134]
Absorption	Graphene	Tapered MMF	DS-DNA	5–400 μM	0.0475 ∆A/μM	<30 s	[135]
Absorption	GO	HB fibre	UV light	2–4 mW	~0.12 ∆A/mW	-	[124]
Absorption	MB-rGO	Tapered SMF	UV light	0.03–12.77 mW	~0.235 dB/mW	-	[125]
Fluorescence	prGO	Etched MMF	Cd^2+^ ions, DA, ssDNA	-	-	-	[142]

^1^ where R is sensor reflectance in %, A is the sensor absorbance in %, and C is the aqueous ethanol concentration in %.
